# Challenges and opportunities in monitoring the long-term well-being of people with HIV in Spain

**DOI:** 10.1371/journal.pone.0325355

**Published:** 2025-08-14

**Authors:** Trenton M. White, María José Fuster-RuizdeApodaca, Carlos Iniesta, Carlos Prats-Silvestre, Asunción Díaz, Aurora Barberá, Jeffrey V. Lazarus

**Affiliations:** 1 Barcelona Institute for Global Health (ISGlobal), Barcelona, Spain; 2 Faculty of Health Sciences, University of Barcelona, Barcelona, Spain; 3 The City University of New York Graduate School of Public Health and Policy (CUNY SPH), New York City, United States of America; 4 The Spanish Interdisciplinary AIDS Society (SEISIDA), Madrid, Spain; 5 Faculty of Psychology, Universidad Nacional de Educación a Distancia (UNED), Madrid, Spain; 6 CIBER de Enfermedades Infecciosas (CIBERINFEC), Instituto de Salud Carlos III, Madrid, Spain; 7 National Center of Epidemiology, Instituto de Salud Carlos III, Madrid, Spain; University of Malaga: Universidad de Malaga, SPAIN

## Abstract

**Background:**

Thanks to antiretroviral therapy, people living with HIV have an increased life expectancy, but face multimorbidity challenges while ageing. This study aimed to evaluate the monitoring capabilities of Spain’s subnational and national health information systems in addressing the multimorbidity needs of people with HIV (PHIV).

**Methods:**

Employing 7 semi-structured focus groups discussions of 20 total participants through purposive sampling of relevant professional profiles recruited by the Spanish Interdisciplinary AIDS Society (SEISIDA), we thematically analysed the discussions using two theoretical frameworks: the World Health Organization health systems building blocks and the DeLone and McLean Information Systems Success Model. Content was validated via participant follow-up and triangulation with publicly available data.

**Results:**

Participant feedback revealed ongoing challenges and capacities within the Spanish health information system for collecting, reporting, and using multimorbidity information among PHIV, including gaps in systematic monitoring of comorbidities, including mental health, technical and interoperability challenges in health data systems, and the need for improved data collection and coordination strategies.

**Conclusion:**

Our findings underscore the importance of a comprehensive approach to health monitoring for chronic care management and the critical role of data standardization and systematization towards improved patient care and public health goal-setting.

## Introduction

The global advancement in HIV treatment, particularly through widespread access to antiretroviral therapy (ART), has greatly improved the life expectancies of people with HIV (PHIV) to nearly match those of the HIV-negative population [1]. Yet, PHIV continue to report lower health-related quality of life (HRQoL) [2–4] and have more comorbidities than their seronegative counterparts, for example, cardiovascular disease, chronic kidney disease, and osteoporosis, which are commonly associated with older age in the general population, as well as infectious diseases such as hepatitis B and hepatitis C [5–7]. Many health systems continue to employ HIV care models that focus predominantly on achieving viral suppression and protecting against opportunistic infections without necessarily focusing on ageing-related issues and long-term well-being, including multimorbidity and HRQoL [8,9].

The HIV “continuum of care” has served as a primary model for monitoring progress in national HIV/AIDS responses since the early 2000s [10]. The Joint United Nations Programme on HIV/AIDS (UNAIDS) targets, the “three 90s” (2014–2020) and “three 95s” (2020–2030) [11], are country targets that have considered three main stages of the care continuum: percentage of PHIV who are diagnosed, who receive ART, and who achieve viral suppression. In 2021, Spain achieved each of the three 90 targets: 92.5%, 96.6% and 90.4%, respectively, with an estimated 80.8% of all PHIV in Spain having reached viral suppression, as per the most recently reported data at the time of submission [12]. A fourth 90 target was proposed in 2016 by HIV researchers suggesting that all PHIV, regardless of viral suppression, should enjoy good HRQoL [13,14]. The fourth 90 emphasizes the importance of not just controlling the virus to undetectable levels but also ensuring that PHIV have a high quality of life and acknowledges the comprehensive well-being of persons. The current World Health Organization (WHO) global strategy for HIV (2022–2030) recognizes the importance of countries measuring stigma, discrimination, and patient-centred approaches to HIV care that achieve a good HRQoL at all stages of the HIV care continuum [15].

Spain’s 17 autonomous communities (regions) are responsible for health system management, including service delivery and the collection of public health and epidemiological data, while the central government maintains competencies for general coordination of public health information [16]. In Spain’s decentralized health system, management of the national health system is coordinated through the Interterritorial Council, which establishes the list of Mandatory Notifiable Diseases [17]. Notification of the epidemiological surveillance for HIV is to occur every three months for new cases and annually for routine monitoring thereafter by the regional surveillance systems to the National Epidemiology Centre. The General Subdirectorate of Health Information of the Ministry of Health is responsible for managing the health information system, which includes the reporting of key indicators through the management of disease registries, and currently does not report indicators for long-term well-being, including multimorbidity and HRQoL [18,19]. Health information system functions are funded centrally through general public taxes, while this funding is managed and allocated at the autonomous community level [16].

Developing national monitoring indicators and strategies for issues beyond the 95-95-95 targets will require deeper understanding of the capacity of and feasibility of health information systems to collect multimorbidity and other well-being data among PHIV. This study aims to examine monitoring capabilities in the subnational and national health information systems of Spain for long-term well-being of PHIV.

## Materials and methods

### Study design and setting

A purposive key informant qualitative study adhering to the Standards for Reporting Qualitative Research (SRQR) [20] was conducted in seven autonomous communities, which were selected for having the highest incidence of new HIV diagnoses in Spain [21]. The seven autonomous communities comprise 72% of Spain’s population [22]. Semi-structured focus groups (n = 7) were held virtually from 19 June to 24 November 2023 among 20 total participants (Table 1) using Microsoft Teams, which allowed for verbatim transcription in Spanish. While the recruitment strategy aimed to include a balanced mix of assistance, public health, and information system experts, representation from public health professionals was more limited than anticipated, despite targeted outreach efforts.

**Table 1:** Focus group dates and participant (n = 20) profiles.

Region of Spain	Date of Focus Group	Participant Profiles		
		Assistance	Information System	Public Health
Andalucia	19 June 2023	3	1	0
Aragon	20 June 2023	2	1	1
Basque country	8 November 2023	1	1	0
Catalonia	19 July 2023	1	1	1
Galicia	22 November 2023	2	1	0
Madrid	24 November 2023	1	1	0
Valencia	24 November 2023	1	1	0

### Participant recruitment

Participants were recruited through purposive sampling from the authors’ professional networks from 14 June 2023 to 21 July 2023, targeting three specific groups: assistance professionals involved in care provision, public health experts, and specialists in healthcare information systems linked with the HIV response. This approach was designed to gain a comprehensive view of the multimorbidity monitoring process across various healthcare sectors.

### Data collection

A quorum of three participants was set for each focus group, where possible, to have sufficient representation to support, question, and resolve any discrepancies that may arise in real-time among individual participants. All focus groups were conducted and recorded in a private Microsoft Teams meeting, with a combination of three researchers from ISGlobal and SEISIDA to direct the focus group using a semi-structured guide, probe unclear statements, and ensure recording quality. The focus group guide (S1 Appendix) was carefully designed by the authors to encourage in-depth discussions about how data related PHIV are collected, managed, reported, and utilized within the Spanish health system. It was based on the present study’s objectives in Spain and informed by relevant literature on health system HIV monitoring and long-term well-being among PLHIV. This design was informed by the authors’ diverse professional expertise in public health, health systems research, and HIV care. Specifically, the authors brought experience in qualitative research methodologies, health information system evaluation, and chronic disease management, which guided the development of targeted questions to explore systemic gaps and opportunities. The guide prompted participants to reflect on their professional experiences with data processes, the challenges they face in integrating multimorbidity indicators into routine HIV monitoring, and their perceptions of system-level solutions. This alignment ensured that the discussions were both comprehensive and focused, generating rich data to address the study’s research aim [20,23]. Prior to implementation, the guide was pilot-tested with two individuals who matched the target participant profiles (one clinical professional and one health information system expert). Their feedback helped refine the wording of questions to ensure clarity and relevance, particularly around technical topics such as data systems. The guide remained consistent across focus groups to facilitate comparability; however, minor adjustments were made during the process to probe emerging themes more deeply as they arose in earlier discussions. Video recordings were transcribed using the Microsoft Teams transcription feature, and both the recordings and transcriptions were saved in password-protected files accessible only to the researchers for subsequent analysis.

The semi-structured guide ensured that each session adhered to a predefined set of topics, maintaining consistency across different regions to gather comprehensive and comparable data. The open-ended questions allowed participants to express their thoughts and experiences in their region. A critical aspect discussed at the beginning of each focus group was the history of the fourth 90, a concept in HIV care focusing on the quality of life for PHIV that emphasizes the importance of going beyond the traditional goals of diagnosis, treatment, and viral suppression [13,14].

### Data analysis

The research team has expertise in public health, HIV care, and health systems research, which informed the study design and analysis. To mitigate potential biases, the team engaged in regular reflexive discussions throughout the study, critically examining how their professional backgrounds and pre-existing knowledge might influence the analysis, while ensuring that findings were grounded in participant perspectives. Transcriptions were thematically analysed to facilitate a comprehensive examination of patterns within the data, which aligned with the study’s theoretical frameworks and permitted an in-depth understanding of the focus group discussions in relation to the study aim [24]. The coding process was conducted in two stages, beginning with the first researcher performing thematic coding by labeling and categorizing key concepts after an initial immersion in the transcripts. This approach facilitated a comprehensive understanding and identification of emergent patterns and themes. In the second stage, coding was performed to classify the extracted text using a thematic analysis within two theoretical frameworks: the six WHO health systems building blocks (i.e., service delivery, health workforce, health information system, access to essential medicines and technologies, financing, and governance) and the six dimensions of the DeLone and McLean Health Information Systems Success Model (i.e., system quality, information quality, service quality, use, user satisfaction, and net benefits) within the health information system building block (S2 Appendix) [20,23]. The researchers characterized the overarching themes that were captured in the coded segments of the focus group discussions, ensuring these characterizations were both reflective of the data and linked to the study aim.

To ensure the validity and reliability of the analysis, several strategies were employed. A two-step coding refinement process was implemented, complemented by a verification system where a second researcher reviewed the coding, thematic development, and findings to maintain objectivity. Discrepancies were resolved by a senior researcher. Participant validation involved sharing key themes and interpretations with focus group participants. Additionally, triangulation with public data from the Ministry of Health and regional health bodies was used to corroborate the findings. This included a systematic review of annual disease surveillance reports and relevant policy documents at both the regional and national levels. Specifically, we examined reports from Spain’s National Epidemiology Centre, regional health departments, and key policy documents such as the Strategic Plan for HIV Prevention and Control (2021–2030). These documents were analyzed to corroborate participant insights on existing monitoring systems, data integration practices, and challenges related to multimorbidity surveillance. By cross-referencing focus group findings with these sources, we ensured consistency and strengthened the validity of the identified themes. This study employed an interpretivist qualitative paradigm, which emphasizes understanding participants’ experiences and perceptions within their specific context. The use of semi-structured focus groups allowed for an in-depth exploration of participant perspectives, while thematic analysis, guided by two theoretical frameworks, provided a structured approach to analyzing and interpreting the data.

### Ethical statement

This study was conducted in full compliance with ethical standards for research involving human participants after approval by the Comité de Ética de la Investigación con medicamentos (CEIm) of Hospital Clinic in Barcelona, Spain, (HCB/2022/0934) in 2023. Informed consent was verbally obtained from all participants, witnessed by their peers and three researchers, and recorded via Microsoft Teams video conferencing after receiving comprehensive information about the study’s purpose, procedures, potential risks, and benefits, and their rights as study subjects.

## Results

**Fig 1:**
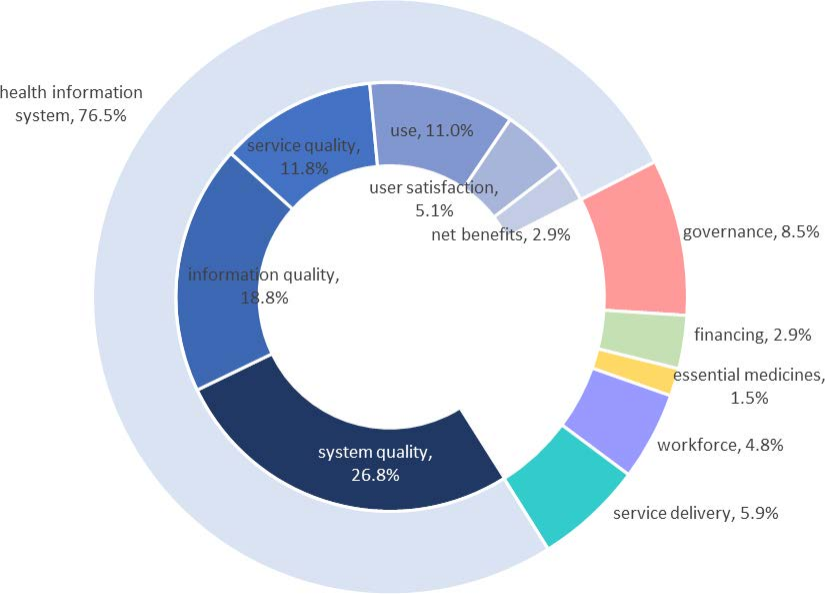
Thematic summary of focus group segments coded by theoretical framework domains. Legend: The outer ring represents the distribution of the 138 segments coded by building blocks of the Health Systems Strengthening (HSS) framework. Within the Health Information System building block, the inner ring represents the dimensions system quality, information quality, service quality, use, user satisfaction, and net benefits of the DeLone and McLean model.

From among 138 coded segments of transcribed focus group text, our analysis revealed substantial capacities and challenges within the Spanish health system to collect, report and use multimorbidity information among PHIV (Fig 1).

### Leadership and governance

Despite frequent discussions on the importance of comorbidities among HIV and infectious disease professionals, participants identified a lack of their integration into systematic monitoring, except for some co-infections like tuberculosis, hepatitis B, and C. HIV and AIDS surveillance systems at both national and autonomous levels are expected to include homogeneous indicators based on case data, deaths, testing, and transmission modes. However, there is no central mandate for indicators measuring multimorbidity or quality of life. Furthermore, participants noted a lack of standardized tools, questionnaires, and guidelines for collecting such data, emphasizing the absence of systematic quality-of-life data collection in healthcare settings, despite ongoing efforts to enhance patient-centered care.

*“The objective of the strategic plan is to improve the quality of life for people with HIV by ensuring non-discrimination, and within that strategic objective, there are various lines of action. The first is to promote the monitoring and incorporation of quality of life measurement in clinical practice.” (Basque country, Public Health)*

### Financing

Several participants indicated that the best methods to collect multimorbidity data might be through clinical trials, cohort studies, or other primary data collection methods, suggesting a reliance on external monitoring and potentially inconsistent financial sources. They also highlighted the need for additional personnel to develop new reporting and surveillance systems or to review medical records, underscoring significant budgetary requirements.

*“It is being paid for by the contacts [participants] through the contributions made in clinical trials.” (Galicia, Assistance)*

### Service delivery

Participants identified multiple challenges in managing comorbidities across chronic conditions, emphasizing the necessity for improved tools and standardized methods, such as a consensus visual form to assess quality of life uniformly across healthcare facilities. They also stressed the need for enhanced training for primary care physicians to better manage specific comorbidities, including diabetes, cardiovascular diseases, mental health, and oncological issues. Despite acknowledging the importance of comorbidities in chronic disease management, participants noted difficulties in systematically incorporating these insights into current monitoring practices.

*“We have thought about this future form, with a visual scale, with a grid, so that it can be done very easily... it’s a project we have, but we haven’t launched it yet.” (Andalusia, Assistance)*

### Health workforce

Healthcare professionals expressed a strong interest in improving care conditions and protocols while recognizing the existing workloads of these professionals, suggesting that any data collection or intervention strategies should be designed with the intent to minimize additional burdens. Limitations of insufficient staffing, multiplicity of systems used in hospitals, and workload in HIV consultations were identified as potentially impacting the capacity to effectively manage and analyse new data from among PHIV.

*“The problem with surveillance is that, after COVID, they have once again reduced staff, and there are not enough people.” (Aragon, Public Health)*

### Access to essential medicines and technologies

Participants discussed the fragmented nature of ART dissemination tracking across autonomous regions, highlighting the challenges due to multiple HIV case recording systems and the reporting of ART and PrEP prescriptions by hospital pharmacies and laboratories without the ability to integrate with other patient data, including epidemiological and diagnostic information. Moreover, some participants saw a potential role for pharmacies in reporting comorbidity treatments, suggesting a shift towards more integrated data collection and management systems. Despite these proposals, including integrated screening and testing services to identify common comorbidities, participants remained uncertain about the impact on the workload of hospital laboratories and information management staff.

### Health information (DeLone and McLean Model)

a *System quality*

Participants noted the use of clinical records to collect data from patients through consultations. Yet the lack of integration of data collection methods for surveillance purposes with clinical practice was identified as a system quality issue. Participants expressed the need for better integration, for example using electronic notifications to remind or prompt physicians and nurses and automated systems to retrieve desired information. The difficulty to cross-reference patient data (e.g., HIV with diabetes) was often acknowledged, attributed sometimes to the lack of systematized collection methods.

*“Each hospital uses a different operating system.” (Aragon, Assistance)*

*“We try to establish the process so that all of this is codified, but the issue has not progressed due to technical problems and other matters... In my center, it may be recorded, but not in a codified or systematic way.” (Catalonia, Assistance)*

Participants noted the fragmentation in hospital and primary care systems, where many chronic health issues are managed, poses challenges to integrating databases. They identified the need for better coordination between clinical and administrative IT departments across regions to create unified systems. The desire for structured, automated data collection was apparent, with suggestions to merge this with existing electronic systems or use regular surveys and cohort studies for specific health conditions. Additionally, two participants questioned the suitability of public health information systems for monitoring, suggesting that primary research via clinical cohorts might be more effective.

*“People go through an HIV consultation, overcrowded, compressed, saturated, so you can’t ask them for much more...” (Andalusia, Assistance)*

b *Information quality*

Participants revealed a lack of systematic and coded data collection for multimorbidity information, with each hospital having its own database(s), which are often not interoperable between hospitals. This would indicate a technical challenge in obtaining coherent and homogeneous information through any potential standardized system for data collection beyond that which is currently collected through quarterly and annual epidemiologic reporting. There is a notable lack of standardization for data collection at the clinical level, leading to potential inconsistencies in data collection and reporting from reviews of clinical notes. This is compounded by the diverse systems used by different entities, which may hinder effective data integration and analysis.

*“The information on HIV is varied, dense, and comes from multiple sources: hospitals, HIV units, epidemiology, pharmacy, and from the population database.” (Aragon, Information Systems)*

c *Service quality*

The potential impact of multimorbidity monitoring on health service quality identified the previously discussed staffing and resource constraints, as well as the lack of integration and interoperability between information management systems, as preventing the use of multimorbidity information to inform public health efforts, such policy planning, at the population level. The use of electronic clinical record notes represents compatibility with healthcare professionals’ workflow and health service delivery. Yet, the repeated emphasis on systematizing data collection and ensuring its clinical relevance reflects the necessity to make data collection purposeful and improvement-driven. Participant assessment of a need for clear criteria on what data should be collected (e.g., which chronic conditions or HRQoL measures) underscore the need for comprehensive health information management to improve services.

*“From the technical difficulties that seemed insurmountable to me, later, when we have worked with engineers and people dedicated to processes and systems, we have found solutions.” (Madrid, Assistance)*

d *Use*

Participants highlighted several challenges, including resource constraints, staff reductions, and the complexities of shifting from passive to active HIV case notification systems. They pointed out critical interoperability issues among hospital data systems, which hinder effective data consolidation and standardization, affecting the use of health information systems across different healthcare settings. This limitation impacts the effective use and collection of patient multimorbidity data. Additionally, technical shortcomings, such as the absence of a structured policy framework for multimorbidity and quality-of-life data collection, along with the reliance on clinicians for case reporting, obstruct the practical use of data in patient care and public health initiatives.

*“Comorbidities are poorly recorded if there is no biological laboratory indicator that makes it easier.” (Catalonia, Information Systems)*

*“The contact for the flow of information, let’s say between hospitals and public health or surveillance, is through the RENAVE [National Surveillance Network] forms.” (Aragon, Information Systems)*

e *User satisfaction*

Despite the identified challenges, an underlying motivation and recognition of the importance of improving system quality was evident among the participants and their impressions of their immediate colleagues. Participants discuss their motivation to address these issues and participate in the development of new plans in improving data collection and integration.

*“Of course, for our part, very motivated just as much as we are, [...] I believe there are no major barriers, although perhaps the only ones may be of a technical nature in terms of time and resources.” (Aragon, Assistance)*

f *Net benefits*

Within this domain emphasizing the health information system’s impact on health, seven segments of text were relevant. The aspirational nature of plans for multimorbidity management is evident. The emphasis on the need for systematic and coded data collection of comorbidities underscores a broader goal rather than a concrete plan. These aspirations indicate a desire to improve health monitoring such that it improves service delivery for chronic care management, including mental health.

*“Because we believe that comorbidities have to be collected, it’s fundamental, not only in the clinical history but in a codified and more systematic way that allows their effectiveness.” (Aragon, Public Health)*

## Discussion

This study set out to examine monitoring capabilities on the long-term well-being of PHIV in the Spanish health information system. Healthcare professionals highlight a substantial gap in the systematic monitoring and integration of comorbidity data in HIV care, which if available could help Spain set national targets beyond viral suppression. They also note the absence of standardized tools for data collection and quality-of-life assessments and suggest enhancing data integration through electronic notifications, automated systems, and better inter-organizational coordination, emphasizing the need for structured, automated collection methods that can simultaneously improve person-centred care and relieve health workforce resource burdens.

New HIV diagnoses data collected within regional health systems must be reported according to surveillance protocols set centrally by the National Surveillance Network (RENAVE) [25]. In addition to new HIV diagnoses collected during epidemiological surveillance, other sources of HIV data reported to the central Ministry of Health include the Hospital Survey of HIV Patients, aggregated reporting of the number of people on ART by the regional governments, and mortality data using mortality statistics from the National Statistics Institute [24]. Aside from new HIV diagnosis, the autonomous regions may elect to collect other data related to each case, but in practice, such systematic collection does not occur for information related to long-term well-being beyond viral suppression, such as multimorbidity or health-related quality of life, according to participants and public reports [25,26]. Objective 4 of the strategic plan 2021–2030 for the prevention and control of HIV and sexually-transmitted infections in Spain, focuses on enhancing the quality of life for PHIV and those at risk of acquiring it [27]. Specifically, it aims to monitor and incorporate quality of life measurements into clinical practice, promote psychosocial health in PHIV, eliminate social and legal barriers, and reduce the stigma associated with HIV.

Identified barriers to multimorbidity data collection, monitoring, and use included a lack of a systematic process for collecting information on comorbidities in existing reporting systems. Participants suggested that a single monitoring program managed by RENAVE that encompasses all PHIV and facilitates consistent and unified data collection would allow for more comprehensive and coherent data to be obtained. Implementing such a program could streamline the data collection process and improve the overall quality of data for analysis and decision-making purposes. Yet, a technical barrier raised by participants included the heterogeneity of comorbidity data that is currently recorded in patient clinical records without an existing mechanism to systematically extract and taxonomise such data.

Clinical guidelines inform HIV clinical practice by establishing uniform standards of evidence-based care, and in Spain are established by the AIDS Study Group (GeSIDA) of the Spanish Society of Infectious Diseases and Clinical Microbiology (SEIMC). Of the current guidelines, six recommend monitoring for co-infection of tuberculosis, hepatitis B, hepatitis C, opportunistic or AIDS-defining infections [28–32]. Participants reported that information on these illnesses are collected by hospitals and, following data quality control procedures, reported in aggregate by the regional disease surveillance entities. Yet, some GeSIDA guidelines also recommend the clinical monitoring of health and well-being indicators beyond viral suppression, for example for kidney and bone diseases [33], neurocognitive morbidity [34] and quality of life [35], which have yet to been systematically monitored by information systems covering PHIV. Our results indicate that these data may be, and often are, recorded by individual physicians in a patient’s clinical record, but are not collated within hospitals or within any region’s health information system. In October 2022, Seville, Spain, was the venue for a global conference of Fast-Track Cities, where the Seville Declaration was endorsed, which emphasizes the critical role local community organizations play in addressing HIV in key urban populations [36], a topic that was not raised in our focus groups, which could indicate a lack of awareness among participants or their perception of a lack of relevance to this topic.

A European study of health information managers in six countries (i.e., Estonia, Italy, the Netherlands, Slovenia, Sweden, and Turkey) regarding their ability to monitor national responses to the HIV epidemic similarly found gaps in the systematic tracking of comorbidities such as bone loss, cardiovascular disease, and neurocognitive disorders, with three of six respondents reporting no monitoring of these aspects [37]. Participants raised the prospective that HIV cohorts can serve as data collection and monitoring mechanisms for issues beyond viral suppression. Sweden has implemented a national HIV registry, InfCareHIV, that covers greater than 99% of PHIV in the country and collects information beyond viral suppression, including long-term comorbidities and patient-reported outcome and experience measures [38]. Similarly, the Netherlands employs a monitoring system through its open national cohort that integrates data from all healthcare settings to monitor comorbidities and healthcare utilisation patterns for greater than 99% of PHIV [39].

This study has several limitations. First, the small sample size and focus group composition, with some groups having only two or three participants, may limit the breadth of perspectives captured. While thematic saturation was achieved, the findings may not fully represent all regional or professional experiences within Spain’s decentralized health system, including the underrepresentation of public health professionals, which may have constrained our ability to fully capture the strategic and population-level perspectives typically contributed by this group. Public health expertise is critical for framing long-term monitoring of well-being beyond individual clinical outcomes, especially in the context of multimorbidity and social determinants of health. Second, participants were recruited through purposive sampling, which, while appropriate for qualitative research, may introduce selection bias and affect the generalizability of the results. Third, as findings are based on participant-reported experiences and perceptions, there is a potential for social desirability bias in responses.

Our findings demonstrate the need for technical and policy improvements to realize the systematic monitoring of comorbidities among specific disease cohorts. There is a clear need for policy frameworks and technical solutions that facilitate the integration of multimorbidity data into health information systems. Such integration could benefit the ability of healthcare providers to offer more holistic and patient-centred care or for policymakers to address health-related quality-of-life concerns at the population level. Furthermore, our results emphasize the role of leadership and governance in driving improvements in health information systems. Effective management and coordination at both national and regional levels are crucial for the successful implementation of standardized and interoperable health data systems. By focusing on multimorbidity and quality of life, alongside traditional HIV care metrics, health systems can provide more comprehensive and effective care, ultimately improving the health outcomes and quality of life for PHIV.

## Supporting information

S1 Appendix Focus group guide. (PDF)

S2 Appendix Description of theoretical analytical frameworks. (PDF)

## References

[1] Bekker LG, Beyrer C, Mgodi N, Lewin SR, Delany-Moretlwe S, Taiwo B. HIV Infection. Nat Rev Dis Primers. 2023;9:1–21. 37591865 10.1038/s41572-023-00452-3

[2] Langebeek N, Kooij KW, Wit FW, Stolte IG, Sprangers MAG, Reiss P. Impact of comorbidity and ageing on health-related quality of life in HIV-positive and HIV-negative individuals. AIDS. 2017;31:1471–81. 28574965 10.1097/QAD.0000000000001511

[3] Engelhard EAN, Smit C, van Dijk PR, Kuijper TM, Wermeling PR, Weel AE, et al. Health-related quality of life of people with HIV: an assessment of patient related factors and comparison with other chronic diseases. AIDS. 2018;32(1):103–12. doi: 10.1097/QAD.0000000000001672 29112062

[4] Degroote S, Vogelaers D, Vandijck DM. What determines health-related quality of life among people living with HIV: an updated review of the literature. Arch Public Health. 2014;72(1):40. doi: 10.1186/2049-3258-72-40 25671112 PMC4323115

[5] Sabin CA, Reiss P. Epidemiology of ageing with HIV: what can we learn from cohorts? AIDS. 2017;31:S121–8. 10.1097/QAD.0000000000001374 28471942

[6] Michaud JM, Zhang T, Shireman TI, Lee Y, Wilson IB. Hazard of cervical, oropharyngeal, and anal cancers in HIV-Infected and HIV-uninfected medicaid beneficiaries. Cancer Epidemiol Biomarkers Prev. 2020;29(7):1447–57. doi: 10.1158/1055-9965.EPI-20-0281 32385117 PMC7334054

[7] Boyd MA, Mocroft A, Ryom L, Monforte A d’Arminio, Sabin C, El-Sadr WM, et al. Cardiovascular disease (CVD) and chronic kidney disease (CKD) event rates in HIV-positive persons at high predicted CVD and CKD risk: A prospective analysis of the D:A:D observational study. PLoS Med. 2017;14(11):e1002424. doi: 10.1371/journal.pmed.1002424 29112958 PMC5675358

[8] Safreed-Harmon K, Anderson J, Azzopardi-Muscat N, Behrens GMN, d’Arminio Monforte A, Davidovich U. Reorienting health systems to care for people with HIV beyond viral suppression. Lancet HIV. 2019;6:e869-77. 10.1016/S2352-3018(19)30334-0 31776099

[9] Lazarus JV, Safreed-Harmon K, Kamarulzaman A, Anderson J, Leite RB, Behrens G. Consensus statement on the role of health systems in advancing the long-term well-being of people living with HIV. Nature Commun. 2021;12:1–14. 34272399 10.1038/s41467-021-24673-w PMC8285468

[10] Gardner EM, McLees MP, Steiner JF, Del Rio C, Burman WJ. The spectrum of engagement in HIV care and its relevance to test-and-treat strategies for prevention of HIV infection. Clin Infectious Dis. 2011;52:793. 10.1093/cid/ciq243 PMC3106261 21367734

[11] Duncombe C, Ravishankar S, Zuniga JM. Fast-Track Cities: striving to end urban HIV epidemics by 2030. Curr Opin HIV AIDS. 2019;14(6):503–8. doi: 10.1097/COH.0000000000000583 31567436

[12] Actualización del continuo de atención del VIH en España, 2021-2022. Madrid: Unidad de vigilancia del VIH ITS y hepatitis B y C; 2023.

[13] Lazarus JV, Safreed-Harmon K, Barton SE, Costagliola D, Dedes N, Del Amo Valero J, et al. Beyond viral suppression of HIV - the new quality of life frontier. BMC Med. 2016;14(1):94. doi: 10.1186/s12916-016-0640-4 27334606 PMC4916540

[14] Harris TG, Rabkin M, El-Sadr WM. Achieving the fourth 90. AIDS. 2018;32:1563–9. 29762172 10.1097/QAD.0000000000001870 PMC6082594

[15] World Health Organization WHO. Global health sector strategies on, respectively, HIV, viral hepatitis and sexually transmitted infections for the period 2022-2030. Geneva: World Health Organization; 2022. https://www.who.int/publications/i/item/9789240053779

[16] Bernal-Delgado E, Garcia-Armesto S, Oliva J, Sanchez Martinez FI, Repullo JR, Pena-Longobardo LM. Spain: health system review. Health Syst Transition. 2018;20:1–179. 30277216

[17] Ministry of Health and Consumption G of S. Royal Decree 2210/1995, of December 28, establishing the national epidemiological surveillance network. BOE. 1996. https://www.boe.es/buscar/act.php?id=BOE-A-1996-1502

[18] Ministry of Health G of S. Annual Report of the National Health System, 2020-21. 2022. https://www.sanidad.gob.es/estadEstudios/estadisticas/sisInfSanSNS/tablasEstadisticas/InfAnSNS.htm

[19] Head of State G of S. Law 16/2003. 2003. https://www.boe.es/buscar/act.php?id=BOE-A-2003-10715

[20] Petter S, DeLone W, McLean E. Measuring information systems success: models, dimensions, measures, and interrelationships. Europ J Inform Syst. 2008;17:236–63.

[21] División de control de VIH y ITS de Epidemiología. Vigilancia epidemiológica del VIH y sida en España 2020: sistema de información sobre nuevos diagnósticos de VIH y registro nacional de casos de sida. Ministerio de Sanidad; 2021. https://www.mscbs.gob.es/ciudadanos/enfLesiones/enfTransmisibles/vih/sida.htm

[22] National Institute for Statistics. Population per autonomous communities and cities and sex. 2020 [Accessed 2024 February 21]. https://www.ine.es/jaxiT3/Tabla.htm?t=2853&L=0

[23] World Health Organization. Monitoring the building blocks of health systems: a handbook of indicators and their measurement strategies. 2010. https://cdn.who.int/media/docs/default-source/service-availability-and-readinessassessment%28sara%29/related-links-%28sara%29/who_mbhss_2010_cover_toc_web.pdf

[24] Braun V, Clarke V. Using thematic analysis in psychology. Qual Res Psychol. 2006;3:77–101.

[25] National Epidemiological Surveillance Network, Carlos III Health Institute, CIBER Epidemiology and Public Health (CIBERESP), Ministry of Economy and Competitiveness, Ministry of Health Social Services and Equality. Protocols of the National Epidemiological Surveillance Network. 2013.

[26] Bernal E, D S, G A J, Fernando O, Sánchez Martínez I, Ramón J. Spain: health system review. Health Syst Transit. 2018. 30277216

[27] Ministry of Health, Government of Spain. Strategic plan for the prevention and control of HIV and STI infections in Spain 2021-2030. 2023.

[28] AIDS Study Group-SEIMC (GeSIDA). GeSIDA Consensus Document for the Evaluation and Treatment of Kidney Diseases in Patients with Human Immunodeficiency Virus (HIV) Infection. 2020. https://gesida-seimc.org/documento-de-consenso-de-gesida-para-la-evaluacion-y-el-tratamiento-de-las-enfermedades-renales-en-pacientes-con-infeccion-por-el-virus-de-la-inmunodeficiencia-humana/

[29] AIDS Study Group-SEIMC. GeSIDA recommendations document on the treatment of tuberculosis in adults infected with Human Immunodeficiency Virus (HIV). 2018. https://gesida-seimc.org/documento-de-recomendaciones-de-gesida-sobre-el-tratamiento-de-la-tuberculosis-en-adultos-infectados-por-el-virus-de-la-inmunodeficiencia-humana/

[30] AIDS Study Group-SEIMC (GeSIDA). Consensus document on the diagnosis and treatment of sexually transmitted infections in adults, children, and adolescents. 2017. https://gesida-seimc.org/wp-content/uploads/2017/06/Documento_de_consenso_sobre_diagnostico_y_tratamiento_de_las_infecciones_de_transmision_sexual_en_adultos_02.pdf

[31] Asociación Española para el Estudio del Hígado, SEIMC. Guías AEEH/SEIMC de manejo de la hepatitis C. 2017. https://seimc.org/contenidos/gruposdeestudio/gehep/dcientificos/documentos/gehep-dc-2017-ManejoHepatitisC-AEEHySEIMC-Marzo.pdf

[32] AIDS Study Group - SEIMC (GeSIDA). Clinical Guide on the Management of Viral Hepatitis in Patients Infected with HIV. 2015. https://www.seimc.org/contenidos/gruposdeestudio/gesida/dcientificos/documentos/2015/gesida-guiasclinicas-2015-Manejo_Hepatitis_Virales.pdf

[33] AIDS Study Group-SEIMC (GeSIDA). Consensus document on osteoporosis and HIV infection. 2016. https://gesida-seimc.org/documento-de-consenso-sobre-la-osteoporosis-en-la-infeccion-por-el-vih-mayo-2016/

[34] AIDS Study Group-SEIMC (GeSIDA). Consensus document on the clinical management of neuropsychiatric and cognitive comorbidity associated with HIV-1 infection. 2020. https://gesida-seimc.org/documento-de-consenso-sobre-el-manejo-clinico-de-la-comorbilidad-neuropsiquiatrica-y-cognitiva-asociada-a-la-infeccion-por-vih-1/

[35] AIDS Study Group-SEIMC (GeSIDA). Shared Management of Patients with HIV Infection between Primary and Hospital Care. 2022. https://gesida-seimc.org/wp-content/uploads/2022/02/manejo-compartido-del-paciente-con-infeccion-por-vih.pdf

[36] UNAIDS. Sevilla declaration on the centrality of affected communities in urban HIV responses unveiled at Fast-Track Cities 2022 conference. 2022 [Accessed 2024 January 16]. https://www.unaids.org/en/resources/presscentre/featurestories/2022/october/20221011_sevilla-declaration-fast-track-cities

[37] Safreed-Harmon K, Kall M, Anderson J, Azzopardi-Muscat N, Behrens GMN, d’Arminio Monforte A. Ability to monitor national responses to the HIV epidemic “beyond viral suppression”: findings from six European countries. Front Public Health. 2020;8:36. 32266194 10.3389/fpubh.2020.00036 PMC7098908

[38] Carlander C, Brännström J, Månsson F, Elvstam O, Albinsson P, Blom S, et al. Cohort profile: InfCareHIV, a prospective registry-based cohort study of people with diagnosed HIV in Sweden. BMJ Open. 2023;13. 10.1136/bmjopen-2022-069688 PMC10030896 36931676

[39] Boender TS, Smit C, Van Sighem A, Bezemer D, Ester CJ, Zaheri S. AIDS therapy evaluation in the Netherlands (ATHENA) national observational HIV cohort: cohort profile. BMJ Open. 2018;8. 10.1136/bmjopen-2018-022516 PMC6169757 30249631

