# Supplementary material for: Challenges and opportunities in monitoring the long-term well-being of people with HIV in Spain
Source: PLoS One. 2025 Aug 14;20(8):e0325355. doi: 10.1371/journal.pone.0325355 (PMC12352655; doi:10.1371/journal.pone.0325355)
Supplement: S1 Appendix — (PDF) [file pone.0325355.s001.pdf]

Supplementary Material 1. Focus group guide, English and Spanish.

ENGLISH

Introduction

Thank you very much for participating in this study

Next, we share with you the script for the focus group that we will have together soon. The purpose of sharing this document with you is so that you can be aware of the questions that will be posed in the focus group and have time to reflect on your answers.

Some of the questions are directed at all participants, regardless of their profile. Others are specifically directed at healthcare professionals, or at administrative healthcare professionals (which includes public health profiles or information systems, registration, or surveillance).

During the in-person session, the moderator will direct the questions to the corresponding profiles, but having this information may help you to understand this script.

Agenda

A. *Welcome and introduction (5-7 minutes)*

B. *Existing monitoring for common comorbidities affecting health-related quality of life (20 minutes)*

1. In the clinical records about your HIV patients, is information collected on the following comorbidities?

	Yes, information is recorded in a coded and systematic manner.	Yes, information is collected in a coded manner, but not systematically.	Yes, information is collected but not in a coded or systematic way.	No information is collected.	I don't know.
1.1 Mental health issues					
1.2 Cardiovascular diseases					
1.3 Neurological and neurodegenerative diseases					
1.4 Drug use or other substance issues					
1.5 Neoplasms, cancers					
1.6 Bone diseases					
1.7 Kidney diseases					
1.8 Pulmonary problems					

1.9 Liver diseases					
1.10 Diabetes					

C. Identification of Barriers to the Collection and Reporting of Comorbidity Data

2. In hospitals, can you identify and describe the potential barriers you face in collecting data on comorbidities in the following areas?

- Technical
- Motivational
- Economic
- Political
- Legal or
- Ethical?

3. Can you identify and describe the potential barriers you face in collecting data on comorbidities in the following areas?

- Technical
- Motivational
- Economic
- Political
- Legal or
- Ethical?

3.1 How do you think the barriers you have identified can be improved or resolved?

3.2 Do you know of any strategic plan, policy, or guideline on the monitoring of comorbidities that you can share?

D. Coordination Among Health System Levels (10 minutes)

4. How do you manage the annual contact between the administration and hospitals to inform and confirm data on HIV?

5. Do you consider it feasible to include some data on comorbidities in your data reporting process? If so, which comorbidities?

E. Monitoring Health-Related Quality of Life in People Living with HIV (PLHIV) (15 minutes)

6. In hospitals, do you use any type of tool or questionnaire to measure the quality of life of people with HIV?

Yes, a tool or questionnaire is used in a coded and systematic manner.	Yes, a tool or questionnaire is used in a coded manner, but not systematically.	Yes, information is collected but not in a coded or systematic way.	No tool or questionnaire is used.	I don't know.
☐	☐	☐	☐	☐

6.1 Why do you use them? What is the motivation for doing so?

6.2 Do you compare the quality of life data of people with HIV with other types of patients who do not have HIV?

6.3 Do you know of any strategic plan, policy, or guideline on monitoring the quality of life of people with HIV that you can share?

7. From the administration or hospitals, do you have any indicators to measure the quality of life of people with HIV?

Yes, we have indicators that are reported.	There are no indicators, but our institution collects these data.	There are no indicators, and our institution does not collect these data.	I don't know.
☐	☐	☐	☐

F. Monitoring issues related to frailty and aging (5 minutes)

8. Do you have specific indicators aimed at addressing chronic conditions, issues related to aging, and frailty (or pre-frailty) in people with HIV? If so, could you indicate which ones?

	Yes, there are indicators.	There are no indicators, but our institution	There are no indicators, and our institution does not	I don't know

		collects these data.	collect these data.	
8.1 Aspects related to aging	☐	☐	☐	☐
8.2 Frailty or pre-frailty	☐	☐	☐	☐

8.3 Do you know of any strategic plans, policies, data, or other guidelines on these aspects (chronicity, aging, and frailty or pre-frailty) that you can share?

G. Final considerations (5 minutes)

ESPAÑOL
Introduccion

Muchas gracias por participar en este estudio.

A continuación, compartimos contigo el guion del grupo focal que tendremos juntos próximamente. El objetivo de compartir contigo este documento es que puedas conocer con antelación las preguntas que serán planteadas en el grupo focal y puedas reflexionar sobre las respuestas.

Algunas de las preguntas van dirigidas a todos los participantes, independientemente de su perfil. Otras van dirigidas específicamente a profesionales sanitarios asistenciales, o a profesionales sanitarios de la administración (lo que incluye los perfiles de salud pública o de sistemas de información, registro o vigilancia).

Durante la sesión presencial el moderador dirigirá las preguntas a los perfiles correspondientes, pero tener esta información puede ayudarte para comprender este guion.

Agenda

A. *Bienvenida y presentación (5-7 minutos)*

B. *Monitoreo existente para comorbilidades comunes que afectan la calidad de vida relacionada con la salud (20 minutos)*

Perfil profesional sanitario asistencial

1. En los registros clínicos sobre tus pacientes con VIH, ¿se recoge información sobre las siguientes comorbilidades?

	Sí, se registra información de manera codificada y sistemática	Sí, se recoge información de manera codificada, pero no sistemáticamente	Sí, se recoge información pero no de manera codificada ni sistemática	No se recoge información	No lo sé
1.1 Problemas de salud mental					
1.2 Enfermedades cardiovasculares					
1.3 Enfermedades neurológicas y neurodegenerativas					

1.4 Problemas de uso de drogas u otras sustancias					
1.5 Neoplasias, canceres					
1.6 Enfermedades oseas					
1.7 Enfermedades renales					
1.8 Problemas pulmonares					
1.9 Enfermedades hepáticas					
1.10 Diabetes					

C. Identificación de barreras para la recopilación y notificación de datos de comorbilidad

2. En los hospitales, ¿podéis identificar y describir las potenciales barreras que tengáis para recoger datos sobre comorbilidades en las siguientes áreas?

- Técnicas
- Motivacionales
- Económicas
- Políticas
- Legales o
- Eticas?

3. ¿Podéis identificar y describir las potenciales barreras que tengáis para recoger datos sobre comorbilidades en las siguientes áreas?

- Técnicas
- Motivacionales
- Económicas
- Políticas
- Legales o
- Eticas?

3.1 ¿Cómo pensáis que se pueden mejorar o resolver las barreras que habéis identificado?

3.2 ¿Conocéis algún plan estratégico, política o guía sobre la monitorización de comorbilidades que podáis compartir?

D. Coordinación entre niveles del sistema de salud (10 minutos)

4. ¿Como tenéis articulado el contacto anual entre la administración y los hospitales para informar y confirmar los datos sobre el VIH?

5. ¿Consideráis factible incluir algunos datos sobre comorbilidades en vuestro proceso de reporte de datos? En caso afirmativo, ¿qué comorbilidades?

E. Monitoreo de la calidad de vida relacionada con la salud en PVVIH (15 minutos)

6. En los hospitales, ¿usáis algún tipo de herramienta o cuestionario para medir la calidad de vida de las personas con VIH?

Si, se usa herramienta o cuestionario de manera codificada y sistemática.	Sí, se usa herramienta o cuestionario de manera codificada, pero no sistemáticamente	Sí, se recoge información pero no de manera codificada ni sistemática	No se usa herramienta o cuestionario.	No lo sé
☐	☐	☐	☐	☐

6.1 ¿Por qué los utilizáis? ¿Cuál es la motivación para ello?

6.2 ¿Comparáis los datos de calidad de vida de las personas con VIH con otros tipos de pacientes que no tienen VIH?

6.3 ¿Conocéis algún plan estratégico, política o guía sobre la monitorización de la calidad de vida de las personas con VIH que podáis compartir?

7. Desde la administración o los hospitales ¿tenéis algún indicador para medir la calidad de vida de las personas con VIH?

Si, tenemos indicadores que se reportan	No existen indicadores, pero en nuestra institución se recogen estos datos	No existen indicadores, y nuestra institución no recoge estos datos	No lo sé
☐	☐	☐	☐

F. Monitoreo de problemas relacionados con la fragilidad y el envejecimiento (5 minutos)

8. ¿Tenéis indicadores específicos dirigidos a abordar la cronicidad, los problemas relacionados con el envejecimiento y la fragilidad (o pre-fragilidad) de las personas con VIH? En caso afirmativo, ¿podrías indicarnos cuáles?

	Si, existen indicadores.	No existen indicadores, pero en nuestra institución se recogen estos datos	No existen indicadores, y nuestra institución no recoge estos datos	No lo sé
8.1 Aspectos relacionados con el envejecimiento	☐	☐	☐	☐
8.2 Fragilidad o pre-fragilidad	☐	☐	☐	☐

8.3 ¿Conocéis algún plan estratégico, políticas, datos u otras guías sobre estos aspectos (cronicidad, envejecimiento y fragilidad o pre-fragilidad) que podáis compartir?

G. Consideraciones finales (5 minutos)
