# Supplementary material for: Challenges and opportunities in monitoring the long-term well-being of people with HIV in Spain
Source: PLoS One. 2025 Aug 14;20(8):e0325355. doi: 10.1371/journal.pone.0325355 (PMC12352655; doi:10.1371/journal.pone.0325355)
Supplement: S2 Appendix — (PDF) [file pone.0325355.s002.pdf]

Supplementary Material 2. Description of theoretical analytical frameworks used

The six health systems building blocks recognised by the World Health Organization are service delivery, health workforce, access to essential medicines and technologies, financing, leadership and governance, and health information [1]. The WHO framework provides a comprehensive view of health systems, encapsulating all critical elements that contribute to their functionality. This comprehensive approach ensures that no aspect of the health system is overlooked in the analysis, making it particularly effective for assessing the system's capability in data monitoring and utilization. The framework's interdependence of the building blocks illustrates how improvements or deficiencies in one area can impact the others. For instance, the quality of data collected (information) is directly influenced by the capacity and motivation of the health workforce, the availability of medical products for diagnostics, and the effectiveness of service delivery. This interconnected view supports a holistic analysis of our qualitative data, helping to identify how issues in data monitoring may be symptomatic of broader systemic challenges.

Specifically within the health information system building block, researchers aimed to thematically and comprehensively analyse participant data. The DeLone and McLean Information Systems Success Model, adapted for health information systems, was selected for its broad applicability to many types of health information systems, including for hospital and national systems [2–5]. This model has the six dimensions of system quality, information quality, service quality, use, user satisfaction, and net benefits [5]. It assesses the effectiveness of information systems by examining both the technical and human factors that influence their success, including the quality of the system and the information it generates, the support services provided to users, and the system's usability and impact on health outcomes. This holistic evaluation is particularly important for assessing the management of information for long-term health conditions. For this study, system quality was understood to refer to the technical reliability, performance, and usability of the health information system. Information quality was understood to refer to the accuracy, relevancy, and timeliness of information collected. Service quality relates to the quality of support the health information system provides to health care delivery. Use covers the intent, extent, and manners in which the health information system is utilized. User satisfaction reflects the attitudes and satisfaction of participants, patients, or other actors and stakeholders within the health information system. The dimension of net benefits was understood to assess the overall positive, negative, or indifferent impacts of the health information system on health service delivery and individual or population health.

References

1. World Health Organization. Monitoring the Building Blocks of Health Systems: A Handbook of Indicators and their Measurement Strategies [Internet]. 2010. Available from: https://cdn.who.int/media/docs/default-source/service-availability-and-readinessassessment%28sara%29/related-links-%28sara%29/who_mbhss_2010_cover_toc_web.pdf

2. Bossen C, Jensen LG, Udsen FW. Evaluation of a comprehensive EHR based on the DeLone and McLean model for IS success: approach, results, and success factors. Int J Med Inform

[Internet]. 2013 [cited 2024 Feb 6];82:940–53. Available from: https://pubmed.ncbi.nlm.nih.gov/23827768/

3. Zheng F, Wang K, Wang Q, Yu T, Wang L, Zhang X, et al. Factors Influencing Clinicians' Use of Hospital Information Systems for Infection Prevention and Control: Cross-Sectional Study Based on the Extended DeLone and McLean Model. J Med Internet Res [Internet]. 2023 [cited 2024 Feb 6];25. Available from: https://pubmed.ncbi.nlm.nih.gov/37347523/

4. Ojo AI. Validation of the DeLone and McLean Information Systems Success Model. Healthc Inform Res [Internet]. 2017 [cited 2024 Feb 6];23:60–6. Available from: https://pubmed.ncbi.nlm.nih.gov/28261532/

5. Petter S, DeLone W, McLean E. Measuring information systems success: models, dimensions, measures, and interrelationships. European Journal of Information Systems. 2008;17:236–63.
